# Management of asthma in pregnant women by general practitioners: A cross sectional survey

**DOI:** 10.1186/1471-2296-12-121

**Published:** 2011-11-03

**Authors:** Angelina S Lim, Kay Stewart, Michael J Abramson, Johnson George

**Affiliations:** 1 Centre for Medicine Use and Safety, Faculty of Pharmacy and Pharmaceutical Sciences, Monash University, Parkville, Victoria, Australia; 2 Department of Epidemiology and Preventive Medicine, Monash University, The Alfred Hospital, Melbourne, Victoria, Australia

## Abstract

**Background:**

Poorly controlled asthma can lead to maternal and fetal complications. Despite the known risks of poorly controlled asthma during pregnancy and the need for stepping up therapy when appropriate, there are concerns that management is suboptimal in primary care.

Our objective was to investigate the management of asthma during pregnancy by general practitioners providing shared maternity care.

**Methods:**

A pre-piloted, anonymous mail survey was sent to all general practitioners (n = 842) involved in shared maternity care at six maternity hospitals in Victoria, Australia. Respondents were asked about their perceived safety of individual asthma medications during pregnancy. Approach to asthma management during pregnancy was further explored using scenarios of pregnant women with stable and deteriorating asthma and poor medication adherence.

**Results:**

Inhaled corticosteroids (ICS) were perceived to be the safest and were the preferred preventive medication in first trimester (74.1%), whilst leukotriene receptor antagonists were the least preferred (2.9%). A quarter (25.8%) of respondents would stop or decrease patients' ICS doses during pregnancy, even when their asthma was well controlled by current therapy. In addition, 12.1% of respondents were not sure how to manage deteriorating asthma during pregnancy and opted to refer to another health professional. Almost half the respondents (48.9%) reported encountering medication nonadherence during pregnancy.

**Conclusion:**

A lack of confidence and/or knowledge among general practitioners in managing deteriorating asthma in pregnancy was observed despite a good understanding of the safety of asthma medications during pregnancy, compliance with evidence-based guidelines in the selection of preventive medications, and self reported good asthma knowledge.

## Background

Optimal asthma control during pregnancy is vital for the well-being of both mother and fetus. Poorly controlled asthma increases the risk of pre-term birth, low birth weight, cesarean section, stillbirth, intrauterine growth restriction (IUGR), congenital malformations (e.g. ventricular and atrial septal defects, spina bifida), small for gestational age (SGA) infants, pre-eclampsia, chorioamnionitis, low APGAR scores and gestational diabetes [1]. Fetal hypoxia, also a result of poorly controlled asthma during pregnancy, can lead to severe risks of neonatal respiratory difficulties, fetal brain ischemia and cerebral palsy [2]. Moreover, fetal growth restriction has been associated with the development of ischemic heart disease, hypertension, and type 2 diabetes in adulthood [3-7]. Conversely, maternal asthma that is well managed has not been associated with any increased risk of complications [8,9]. A promptly treated acute asthma attack during pregnancy is unlikely to have a serious effect on the pregnancy, delivery, or the health of the infant [10].

A decrease in the use of inhaled anti-inflammatory agents (preventers) [inhaled corticosteroids (ICS)], symptom controllers [long-acting beta agonists (LABA)] and their combinations, but an increase in the use of relievers [short-acting beta_2 _agonists (SABA)] during pregnancy has been reported [11]. Chambers found that two in five women discontinued or reduced their asthma medication during pregnancy, leaving them at risk of uncontrolled asthma [12]. Women who decreased their ICS medication have been shown to deliver offspring with lower mean birth weight and length than women who did not [13].

Asthma management during pregnancy should follow the stepwise management of asthma in adults. The British Thoracic Society (BTS),[14] Global Initiative for Asthma (GINA),[15] National Asthma Council of Australia (NAC)[16] and National Heart, Lung and Blood Institute (NHLBI)[17] recommend continuing pregnant women on the same asthma therapy used prior to the pregnancy, if their asthma is well controlled on that regimen. ICS are the recommended first line agents for the treatment of mild to moderate persistent asthma in most guidelines except those of the American College of Obstetricians and Gynecologists and the American College of Allergy, Asthma and Immunology (ACOG & ACAAI),[18] which recommend cromones ahead of ICS. A switch to budesonide is recommended for women who are planning a pregnancy and already using an ICS, as it has more evidence for safety during pregnancy [16].

Even though evidence is lacking for the safety of some asthma medications during pregnancy, the risks of uncontrolled asthma during pregnancy are very clear. The authors recently conducted a systematic review of the safety of regular preventive asthma medications during pregnancy and reported some negative outcomes, but also noted many factors confounding the trials [19]. It was concluded that asthma preventive medications did not cause poor perinatal outcomes. Health care providers should not hesitate to increase doses or introduce additional medications as needed. Selection of preventive medications for asthma management during pregnancy should be based on an assessment of the risks and benefits of medication use versus the risks of poorly controlled asthma [19].

The main barrier reported when prescribing in pregnancy was access to current information about medication effects on the foetus, due to lack of a single comprehensive source of information, lack of time to access information and rapidly outdated information [20]. Physicians' and obstetricians' heavy reliance on the United States Food and Drug Administration (FDA) pregnancy risk categories, in combination with their reluctance to weigh risks versus benefits of medication use in pregnancy in individual patients, may be contributing to low prescribing rates during pregnancy [20]. In one study from the United States, pregnant asthmatic women were significantly less likely to be prescribed oral steroids either in the emergency department or on discharge from hospital than were non-pregnant asthmatic women [11]. The pregnant women were also three times more likely than non-pregnant women to report ongoing asthma exacerbations following hospital discharge [11].

There is little information available regarding prescribing trends in pregnant women with asthma in primary care. Despite the overwhelming consensus that pregnant women with asthma should be rigorously managed, doctors still under-prescribe. Due to the increased risk of poor perinatal outcomes, pregnant asthmatic women are recommended to have their asthma monitored at least once a month and their therapy should be increased when appropriate [17]. A study from Yale University found that two-thirds of pregnant asthmatic women were under-treated for asthma for three or more months of pregnancy [12]. Pregnant women were concerned about using steroids during pregnancy due to the perceived effects on the unborn child. Two in five women said they would be more likely to continue taking their asthma medication during pregnancy if their prescriber had recommended it, showing that prescribers have a vital role in encouraging patient adherence to treatment recommendations. Understanding the prescribing practices of physicians providing care to pregnant women with asthma would provide valuable information to optimise asthma management during pregnancy.

The objective of this study was to describe the management of asthma during pregnancy by general practitioners, with a view to informing initiatives for improving asthma management, leading to improved maternal and fetal outcomes.

## Methods

An anonymous mail questionnaire was sent to all general practitioners involved in shared maternity care (n = 842) at six public maternity hospitals in Melbourne, Australia. Shared care general practitioners are affiliated with one or more maternity hospitals and review women with uncomplicated pregnancies regularly until delivery, rather than having them attend maternity hospital out-patient antenatal clinics. Any general practitioner, who is accredited at a maternity hospital as a Shared Maternity Care Affiliate, can provide maternity shared care. To find out more about this model of care and the accreditation processes, please refer to http://www.health.vic.gov.au/maternitycare/smcaguidelines.pdf

The questionnaire was designed by the authors using items derived from the literature and discussion with pharmacists and respiratory consultants. Face and content validity was established through review by other experienced researchers (n = 13) and general practitioners (n = 4). Minimal changes were made to the questionnaire based on feedback. The final questionnaire (see additional file 1) consisted of three sections: The first section had 9 items on demographics and the prescriber's practice. The second section asked respondents to rank their preferences for prescribing preventive asthma medication during pregnancy and to indicate their perceived safety of different asthma medications in each trimester. The third section aimed to gauge respondents' likely approach to asthma management in pregnant women with the help of two scenarios.

Initially, an explanatory letter, questionnaire and reply-paid envelope were mailed by administrative staff in charge of shared maternity care at each hospital. Due to the anonymous nature of the survey, a blanket reminder letter, questionnaire and reply-paid envelope were sent to all general practitioners six weeks later. As an incentive, potential participants were advised that a small donation would be made to the Asthma Foundation of Victoria for each completed survey returned to the investigators.

This study was endorsed by The Asthma Foundation of Victoria and approved by the Monash University Human Research Ethics Committee (Approval no. CF10/2750 - 2010001557). Permission to contact participants was also sought from all participating institutions.

### Statistical analysis

For a population of 850 general practitioners, 144 responses were required to ensure that the sample proportions would be within ±5% of the true values with a 90% level of confidence. Data were analysed using SPSS, version 19.0 (IBM, Somers, NY, USA, 2010). Chi square and independent sample t-tests were performed to investigate relationships between prescriber demographics and prescribing appropriateness, which was identified for the vignettes according to global asthma guidelines [14-18]. Significance level was set at P < 0.05.

## Results

A total of 176 questionnaires were returned (response rate 20.9%); two were excluded from the analysis as more than 20% of items were unanswered. Respondents were mostly female (70.7%), practising in the metropolitan region (84.5%) and had practised as general practitioners for a median of 19 years. Approximately one-third of respondents had encountered asthma in more than 10% of their pregnant patients. The characteristics of respondents are shown in Table 1.

**Table 1:** Demographics and practice information of respondents (n = 174)*

Characteristic	n (%)
**Gender**	
Female	123 (70.7%)
**Years of practice as a Family Practitioner**	

≤10years	23 (13.2%)
11-20years	63 (36.1%)
21-30years	63 (36.1%)
>30years	24 (13.7%)

**Current practice location**	

Metropolitan	147 (84.5%)
Regional	15 (8.6%)
Rural	11 (6.3%)

**Proportion of pregnant women cared for who have asthma**	

None	6 (3.4%)
<10%	108 (62.1%)
11-20%	52 (29.9%)
>20%	4 (2.3%)

**Assistance with asthma management**	

None	120 (69.0%)
Practice nurse	34 (19.5%)
Asthma educator	4 (2.3%)
Both an asthma educator and practice nurse	3 (1.7%)

**Perceived asthma knowledge**	

Poor	1 (0.6%)
Average	56 (32.2%)
Good	81 (46.6%)
Very good	35 (20.1%)

*Some numbers do not add up to 174 due to missing data

ICS (74.1%) were the preferred preventive medication for a pregnant woman with worsening asthma in the first trimester, while leukotriene receptor antagonists (LTRA) (2.9%) were the least preferred agents (Table 2). At normal adult doses, ICS and beta_2 _agonists (short- and long-acting) were perceived to be safe in all trimesters by the majority of respondents, while participants had concerns about the safety of LTRA during all trimesters (Table 3).

**Table 2:** Preferences for asthma preventive medication use in pregnancy (n = 174)

Asthma preventive medication class	n (%)	
	**First preference**	**Second preference**
	
**Cromones**	13 (7.5%)	22 (12.6%)
**Leukotriene receptor antagonists (LTRA)**	5 (2.9%)	3 (1.7%)
**Inhaled corticosteroids (ICS)**	129 (74.1%)	24 (13.8%)
**Long-acting beta_2 _agonists (LABA)**	10 (5.7%)	11 (6.3%)
**LABA/ICS combination**	36 (20.7%)	73 (42.0%)

*Some numbers do not add up to 174 due to missing data

**Table 3:** Perceived safety of asthma medications during pregnancy in different trimesters (n = 174)

	n (%)					
	**First trimester**		**Second trimester**		**Third trimester**	

**Drug**	**Yes**	**No**	**Yes**	**No**	**Yes**	**No**

**Cromones**						

Nedocromil	88 (50.9%)	41 (23.6%)	102 (59.0%)	22 (12.6%)	100 (57.8%)	23 (13.2%)
Sodium Cromoglycate	112 (64.4%)	29 (16.7%)	125 (71.8%)	15 (8.6%)	123 (70.7%)	17 (9.8%)

**Inhaled corticosteroids**						

Beclomethasone	132 (75.9%)	23 (13.2%)	140 (80.5%)	16 (9.2%)	142 (81.6%)	15 (8.6%)
Budesonide	154 (88.5%)	6 (3.4%)	159 (91.4%)	3 (1.7%)	156 (89.7%)	3 (1.7%)
Ciclesonide	111 (63.8%)	31 (17.8%)	120 (69.0%)	23 (13.2%)	121 (69.5%)	21 (12.1%)
Fluticasone	133 (76.4%)	26 (14.9%)	144 (82.8%)	14 (8.0%)	147 (84.5%)	11 (6.3%)

**Leukotriene receptor antagonists**						

Montelukast	47 (27.0%)	79 (45.4%)	60 (34.5%)	67 (38.5%)	59 (33.9%)	68 (39.1%)
Zafirlukast	29 (16.7%)	83 (47.7%)	40 (23.0%)	73 (42.0%)	40 (23.0%)	73 (42.0%)

**Long-acting beta_2 _agonists**						

Eformoterol	101 (58.0%)	46 (26.4%)	120 (69.0%)	21 (12.1%)	121 (69.5%)	23 (13.2%)
Salmeterol	105 (60.3%)	44 (25.3%)	124 (71.3%)	20 (11.5%)	125 (71.8%)	22 (12.6%)

**Oral corticosteroids**						

Prednisolone	141 (81.0%)	20 (11.5%)	151 (86.8%)	8 (4.6%)	149 (85.6%)	12 (6.9%)

**Short-acting beta_2 _agonists**						

Salbutamol	167 (96.0%)	0 (0.0%)	164 (94.3%)	0 (0.0%)	164 (94.3%)	1 (0.6%)
Terbutaline	146 (83.9%)	5 (2.9%)	144 (82.8%)	4 (2.3%)	144 (82.8%)	6 (3.4%)

*Some numbers do not add up to 174 due to missing data

### Preferred management of asthma in pregnant women

#### Case vignette one part one (stable asthma in pregnancy)

A quarter of respondents would stop or reduce the dosage of preventive asthma medication during pregnancy, even though the patient's asthma was well controlled on the regimen prior to pregnancy (Table 4). A single agent therapy rather than the combination was preferred by 20.3% of respondents; but one respondent preferred to use ICS alone. A few (4.0%) decided to decrease the dose of the salmeterol/fluticasone combination.

**Table 4:** Responses for case vignette one (n = 174)

Case vignette one: A patient of yours has recently become pregnant. She has moderate asthma which is well controlled with **salmeterol/fluticasone (250/25)**, one puff twice daily, and salbutamol inhaler as required. She has no other medical conditions nor is she taking any other medications. **Part one (Stable asthma in pregnancy): **She wonders whether she should continue these medications during pregnancy. What is your intended action?	
**Responses for (i)**	**n (%)**

Continue her on the same medications	123 (70.7%)
Decrease her dose	43 (24.7%)
Refer	5 (2.9%)
Stop her medication	2 (1.2%)
**Part two (deterioating asthma in pregnancy): **A few weeks pass by and your patient returns. You notice that her asthma is deteriorating. She tells you that she has been using her salbutamol inhaler more than three times per week. She has been compliant with the salmeterol/fluticasone and has had no changes to her asthma medication regimen nor has she had any changes in lifestyle. What is your intended action?	

**Responses for (ii)**	**n (%)**

Increase her dose	116 (66.7%)
Refer	21 (12.1%)
Continue her on the same regimen and just monitor her asthma more closely	15 (8.6%)
Add another agent	10 (5.7%)
Decrease ICS regimen	9 (5.2%)

*Some numbers do not add up to 174 due to missing data

#### Case vignette one part two (deteriorating asthma in pregnancy)

Only 62.6% of general practitioners opted to increase the dosage of the current regimen on deterioration of symptoms (Table 4). A few respondents (4.6%) decided to continue the same regimen and not intervene, leaving the patient at risk of uncontrolled asthma.

Patterns of prescribing and management had no association with the proportion of pregnant women treated per year, years in practice, clinical setting, the availability of help from a practice nurse or asthma educator, perceived knowledge, or asthma management guidelines followed (P values all greater than 0.05).

#### Case vignette two (nonadherent asthmatic pregnant patient)

over three quarters of respondents (82.2%) would reinforce the need to use the eformoterol/budesonide combination by the 18 weeks pregnant woman with concerns about the safety of this combination, given her history of poor adherence (Table 5). Only two respondents chose not to reinforce the need to adhere to the asthma regimen and a few (12.4%) chose to switch her to another preventive medication that allowed more convenient dosing.

**Table 5:** Responses for case vignette two (n = 174)

Case vignette two One of your regular patients is 18 weeks pregnant and she asks you for a new prescription for salbutamol inhaler, as she is a health care card holder and can get them cheaper on script. However, you notice that she got a script for salbutamol inhaler only last month. Upon asking, you find out that she has stopped her budesonide/eformoterol inhaler because she fears it will harm her unborn child. Instead she has been using her salbutamol inhaler more frequently to compensate. She has no other medical conditions nor is she taking any other medications.	
(i) **Part one (nonadherent asthmatic pregnant patient): **What is your intended action?*	
*Participants could only tick one response for part one	
**Responses for (i)**	**n (%)**

Give script for salbutamol and reinforce the need to continue her preventive medication	143 (82.2%)
Give script for salbutamol but change her onto a different preventive medication	21 (12.4%)
Give script for salbutamol and refer	6 (3.5%)
Give script for salbutamol with no further questions	2 (1.2%)
Do not give the salbutamol script and refer patient	0 (0.0%)

*Some numbers do not add up to 174 due to missing data

Almost half of the respondents (48.9%) reported encountering patients with poor adherence to preventive asthma medications during pregnancy, putting them at risk of complications. Strategies they employed for improving adherence were: providing education on risks associated with nonadherence to asthma medications during pregnancy and poor asthma control (46.6%); providing education focusing on the safety of asthma medications during pregnancy (42.0%); organising regular visits to monitor asthma control (36.2%); organising regular return visits to monitor adherence (27.6%); referral to another health professional to monitor asthma control (8.6%) and referral to another health professional to monitor adherence (4.6%).

## Discussion

This study has shown a strong preference for ICS as first line preventive therapy, which is the recommended agent for pregnant women by most guidelines, including the NAC guidelines [14-17]. In reporting perceived safety of asthma medications in each trimester, ICS were regarded as safe throughout pregnancy. Uncertainty about the safety of LTRA throughout pregnancy was evident. This could possibly be attributed to limited safety data available on these newer medications and/or prescribers' lesser familiarity with these drugs.

It is comforting to know that prescribers are confident addressing poor adherence when confronted with the situation; all respondents listed strategies they have used to improve adherence when noncompliance was encountered. However, it is well known that patients do not normally admit nonadherence and prescribers rarely ask about adherence during consultations [21].

The majority of respondents opted to keep the woman who recently became pregnant on the same asthma regimen as that prior to conception. However, a considerable number of prescribers would either decrease or stop her medication or refer her to another health professional, even though her asthma was well controlled on a fluticasone/salmeterol regimen. Not surprisingly, more prescribers opted to refer when her asthma started deteriorating. Although referral was not an inappropriate action, the case presented did not warrant referral according to the Asthma Management Guidlelines[22] e.g. life threatening asthma attacks, no response to therapy, need for frequent courses of oral corticosteroids. This suggests a lack of confidence and/or knowledge among general practitioners in managing deteriorating asthma in pregnancy. Prescribers apparently feel more comfortable referring these patients; this is surprising in light of the fact that two-thirds of respondents rated their asthma knowledge as good or very good.

There were no differences between experienced and less experienced prescribers in the appropriateness of asthma medicines selected. This is evidence against the suggestion that less experienced prescribers would be more likely to under-prescribe and deem medications unsafe in pregnancy [23]. The proportion of pregnant women treated per year, the clinical setting, support from a practice nurse or asthma educator, perceived knowledge, and use of guidelines were not found to predict appropriate prescribing, but interpretation was limited by low sample size. Some prescribers who said they followed a particular guideline for asthma management still commented that they would like more information regarding management for this population. Morgan *et al*. found that the major barriers to prescribing during pregnancy were lack of a single comprehensive source of information, lack of time and the fact that information gets outdated rapidly [23].

Limited data were available on the study population, but most characteristics were in line with the general population of Australian general practitioners, with the majority practicing in the metropolitan area (84.5% vs. 71% as the national average in 2009-10) [24]. No statistics are available on the characteristics of general practitioners involved in shared maternity care. Our sample had a considerably higher proportion of females than in the Australian population of practicing general practitioners (70.7% vs 44%);[24] shared maternity care may be more attractive to female general practitioners. Our respondents were more likely to have a nurse in their practice than reported in the national statistics (21.2% vs 9.0%);[24] however, patients attending nurse-run asthma clinics based in Australian general practice did not show a greater improvement in quality of life or lung function compared with those receiving usual care [25].

### Strengths and limitations

This study has provided information about general practitioners' likely management of asthma in pregnant women. To the best of our knowledge, a survey of this nature has not been previously reported. The study population comprised general practitioners providing shared maternity care in affiliation with all the major Victorian maternity hospitals, including the largest maternity hospital in Australia. Our findings should make general practitioners more aware of under utilization of preventive asthma medications during pregnancy and improve the management of women with asthma.

Our study had a modest response rate; however, this level of response is typical for a postal survey directed to general practitioners [26]. As the study was anonymous, we were unable to follow up the non-respondents to improve the response rate or to compare their characteristics with those of the respondents. Responses were received from general practitioners with a range of characteristics; nevertheless, it is possible that the respondents were more knowledgeable than their counterparts, in which case the extent of poor practice observed would be an underestimation. Thus, it is possible that the perception of general practitioners and poor management of pregnant asthmatic women could be underestimated. There is limited information on women's experiences of asthma management during pregnancy; however, the authors are currently conducting a qualitative study with pregnant asthmatic women in each trimester and with varying asthma severity to address this gap in the literature.

The authors are developing interventions and strategies to promote awareness of poor asthma management during pregnancy targeting both pregnant women with asthma and their health professionals. These interventions may take the form of educational modules that can be used for continuing education of health care providers, or antenatal clinics specifically for pregnant women with asthma.

## Conclusion

Overall, general practitioners had a good understanding of the safety of asthma medications during pregnancy, complied with evidence-based guidelines in the selection of preventive medications, and self reported good asthma knowledge. Despite this, a lack of confidence and/or knowledge among general practitioners in managing deteriorating asthma in pregnancy was observed. The findings from this survey will inform the development of future interventions and strategies to optimize asthma management and outcomes in pregnant women.

## List of abbreviations

ACOG & ACAAI: American College of Obstetricians and Gynecologists & the American College of Allergy; Asthma and Immunology; APGAR: Named after Dr Viginia Apgar (calculated by scores of the newborn's activity; pulse; grimace; appearance; respiration usually at 1 to 5 minutes after delivery); BTS: British Thoracic Society; FDA: Food and Drug Administration; ICS: Inhaled corticosteroids; IUGR: Intrauterine growth restriction; GINA: Global Initiative for Asthma; LABA: Long-acting beta_2 _agonists; LTRA: Leukotriene receptor antagonists; NAC: National Asthma Council of Australia; NHLBI: National Heart; Lung and Blood Institute; SABA: Short-acting beta_2 _agonist; SGA: Small for gestational age.

## Competing interests

The authors declare that they have no competing interests

Conflicts of interest: Angelina Lim - none, Kay Stewart - none, Michael Abramson - was a member of the scientific committee for a meeting sponsored by GlaxoSmithKline, Johnson George - none

This project was endorsed but not funded by the Asthma Foundation of Victoria.

## Authors' contributions

AL proposed the original concept and developed it with input from JG, KS and MA. AL was responsible for data collection and entry. All authors were involved in data analysis, interpretation and manuscript preparation. All authors approved the final manuscript.

## Pre-publication history

The pre-publication history for this paper can be accessed here:

http://www.biomedcentral.com/1471-2296/12/121/prepub

## Supplementary Material

Additional file 1 **Management of pregnant women with asthma survey**. This is the questionnaire used in our study to investigate prescribing patterns and management strategies of pregnant women with asthma by general practitioners. This survey was given to all our participants and endorsed by the Asthma Foundation of Victoria. Click here for file
