# Supplementary material for: Management of asthma in pregnant women by general practitioners: A cross sectional survey
Source: BMC Fam Pract. 2011 Nov 3;12:121. doi: 10.1186/1471-2296-12-121 (PMC3219736; doi:10.1186/1471-2296-12-121)
Supplement: Additional file 1 — Management of pregnant women with asthma survey. This is the questionnaire used in our study to investigate prescribing patterns and management strategies of pregnant women with asthma by general practitioners. This survey was given to all our participants and endorsed by the Asthma Foundation of Victoria. [file 1471-2296-12-121-S1.PDF]

Section one: You and your practice

The questions in this section are about you and your practice. Please either fill in the gaps or tick (✓) the boxes as appropriate.

1. **Gender:** ☐ MALE ☐ FEMALE

2. **The year you began practising as a General Practitioner** ☐☐☐☐

3. **Did you complete ALL your medical training in Australia?**

Please tick (✓) one (1) box only

☐ Yes

☐ No, Please specify where____

(Specify if more than one location is applicable i.e. Australia/United Kingdom)

If NO, how long have you been practising in Australia? ______years

4. Do you have any areas of expertise (e.g. asthma, women's health, paediatrics)?

Please tick (✓) one (1) box only

☐ Yes, please specify ____

☐ No

5. Your practice setting could be described as? *Please tick (✓) one (1) box only*

☐ Metropolitan ☐ Regional ☐ Rural

6a. On average, how many pregnant women do you provide shared care in a year?

Please tick (✓) one (1) box only

☐ None (go to question 7) ☐ <10 ☐ 11-20 ☐ >20

6b. Approximately, what proportion of these pregnant women have asthma?

Please tick (✓) one (1) box only

☐ None ☐ <10% ☐ 11-20% ☐ 20-30% ☐ >30%

7. Do you have a practice nurse or asthma educator who assists you in asthma management?

Please tick (✓) one (1) box only

☐ Yes, please specify _______________________________________ ☐ No

8. Do you follow any guideline for managing asthma? *Please tick (✓) one (1) box only*

☐ Yes, please specify _______________________________________ ☐ No

9. On a scale of very poor to very good, how would you rate your knowledge in managing asthma?

Please tick (✓) one (1) box only

☐ Very poor ☐ Poor ☐ Average ☐ Good ☐ Very Good

Section two: Safety of asthma medications during pregnancy

The two questions in this section are to investigate the perceived safety of different asthma medications during pregnancy.

10. You need to prescribe a PREVENTIVE medication to a pregnant patient with worsening asthma who is in her <u>FIRST TRIMESTER</u>. She has no other medical conditions nor is taking any other medications. Give your top two preferences. *(Put a **"1"** in the box of your first preference, and a **"2"** in the box of your second preference. You can have more than one 1st OR 2nd preference)*

- ☐ Cromolyns (nedocromil, sodium cromoglycate)
- ☐ Inhaled corticosteroids (beclomethasone, budesonide, ciclesonide, fluticasone)
- ☐ Leukotriene receptor antagonists (montelukast, zafirlukast)
- ☐ Long-acting beta$_2$ agonists (eformoterol, salmeterol)
- ☐ Long-acting beta$_2$ agonists + inhaled corticosteroid combination (Seretide, Symbicort)

11. At normal adult doses, which of the following asthma medicines do you consider 'safe' for use in a pregnant woman with asthma? *Please tick (✓) either "Yes" or "No" for each drug for each trimester.*

Example:

Drug	First trimester		Second trimester		Third trimester	
	Yes	No	Yes	No	Yes	No
Drug A	✓			✓		✓

Now complete the following table

Drug	First trimester		Second trimester		Third trimester	
	Yes	No	Yes	No	Yes	No
Cromolyns						
Nedocromil						
Sodium Cromoglycate						
Inhaled corticosteroids						
Beclomethasone						
Budesonide						
Ciclesonide						
Fluticasone						
Leukotriene receptor antagonists						
Montelukast						
Zafirlukast						
Long acting beta$_2$ agonists						
Eformoterol						
Salmeterol						
Oral corticosteroids						
Prednisolone						
Short acting beta$_2$ agonists						
Salbutamol						
Terbutaline						

Section three: Management of pregnant women with asthma

This section has two (2) scenarios of pregnant women with asthma. Each scenario is accompanied by some questions.

SCENARIO ONE:

Refer to the following scenario and answer questions 9 and 10

A patient of yours has recently become pregnant. She has moderate asthma which is well controlled with ***Seretide (250/25)*** *(fluticasone /salmeterol) one puff twice daily and Ventolin (Salbutamol) Inhaler as required. She has no other medical conditions nor is she taking any other medications.*

12. She wonders whether she should continue these medications during pregnancy. What is your intended action? *Please tick (✓) one (1) box only*

☐ Continue her on the same medications

☐ Change Seretide to Pulmicort (Budesonide)

☐ Change Seretide to Symbicort (Budesonide/eformoterol)

☐ Change her to a different preventive medication(s), specify which one(s)___________________________________

☐ Stop Seretide

☐ Decrease the dose of Seretide

☐ Refer her to another health professional (e.g. Respiratory specialist)

☐ Other, please specify

____

13. A few weeks pass by and your patient returns, and you notice that her asthma is deteriorating. She tells you that she has been using her Ventolin inhaler more than three times per week. She has been compliant with the Seretide and has had no changes to her asthma medication regimen nor has she had any changes in lifestyle. What is your intended action? *Please tick (✓) one (1) box only*

☐ Increase the dose of Seretide

☐ Continue with the same regimen and simply monitor her asthma more closely

☐ Change her to another preventive medication, specify which one(s)

____

☐ Add another preventive medication, specify which one(s)____

☐ Refer her to another health professional (e.g. Respiratory specialist, obstetrician)

☐ Other, please specify

____

____

SCENARIO TWO:

Refer to the following scenario and answer questions 11 and 12

> *One of your regular patients is **18 weeks pregnant** and she **asks you for a new prescription of Ventolin inhaler** as she is a health care card holder and can get them cheaper on script. However, you notice that she got a script of **Ventolin Inhaler only last month**. Upon asking, you find out that she has stopped her **Symbicort** (budesonide/eformeterol) inhaler because she fears it will harm her unborn child. Instead she has been using her Ventolin inhaler more frequently to compensate. She has no other medical conditions nor is she taking any other medications.*

14. What is your intended action? *Please tick (✓) one (1) box only*

☐ DO NOT give a script for the Ventolin inhaler, but refer her to a respiratory specialist

☐ Give a script for Ventolin Inhaler and refer her to a respiratory specialist

☐ Give a script for Ventolin Inhaler with no further questions and just monitor her asthma more closely thereafter

☐ Give a script for Ventolin Inhaler, discuss the importance and safety of Symbicort and reinforce the need for her to continue Symbicort

☐ Give a script for Ventolin Inhaler and initiate her on a different preventer, specify which one(s)
____

☐ Other, specify____

15a. Have you ever had to intervene and promote compliance (adherence) to preventive asthma medication(s) in a noncompliant (non-adherent) patient during pregnancy? *Please tick (✓) one (1) box only*

☐ Yes ☞ please go on to 12 b ☐ No

15b. If *yes*, which of the following strategies have you employed? *Please tick (✓) as many boxes as appropriate*

☐ Provided education focusing on the safety of asthma medications

☐ Provided education on the risks associated with non-adherence to asthma medications during pregnancy and poor asthma control

☐ Organised regular return visits to monitor adherence to asthma medications

☐ Organised regular return visits to monitor asthma control

☐ Referred them to another health professional (e.g. pharmacist, asthma educator) to monitor their adherence

☐ Referred them to another health professional (e.g. respiratory specialist) to monitor their asthma control

☐ Other, please specify ____
____

Thank you for your time!

Send the completed questionnaire in the reply-paid envelope provided to Monash University, Parkville.
